# Prognostic value of plasminogen activator inhibitor‐1 in biomarker exploration using multiplex immunoassay in patients with metastatic renal cell carcinoma treated with axitinib

**DOI:** 10.1002/hsr2.197

**Published:** 2020-10-15

**Authors:** Naoko Honma, Takamitsu Inoue, Norihiko Tsuchiya, Atsushi Koizumi, Ryohei Yamamoto, Taketoshi Nara, Sohei Kanda, Mingguo Huang, Kazuyuki Numakura, Mitsuru Saito, Shintaro Narita, Shigeru Satoh, Tomonori Habuchi

**Affiliations:** ^1^ Department of Urology Akita University Graduate School of Medicine Akita Japan; ^2^ AMED‐CREST Japan Science and Technology Agency Tokyo Japan; ^3^ Department of Urology Yamagata University Faculty of Medicine Yamagata Japan; ^4^ Center for Kidney Disease and Transplantation Akita University Hospital Akita Japan

**Keywords:** metastatic renal cell carcinoma, molecular‐targeted therapy, plasminogen activator inhibitor‐1, serum biomarker

## Abstract

**Background and Aims:**

Vascular endothelial growth factor‐directed therapies play a significant role in patients with metastatic renal cell carcinoma (mRCC). Biomarkers for predicting treatment efficacy and resistance are required to develop personalized medicine. We evaluated multiple serum cytokine levels in patients with mRCC treated with axitinib to explore predictive biomarkers.

**Methods:**

From September 2012 to October 2015, serum samples were collected from 44 patients with mRCC before treatment and 4 weeks after axitinib initiation. Bio‐Plex Pro Human Cancer Biomarker Panels 1 and 2 were used to measure levels of 34 serum biomarkers related to angiogenesis and cell proliferation.

**Results:**

Patients with partial response or stable disease had significantly decreased serum plasminogen activator inhibitor‐1 (PAI‐1) level from pre‐treatment to 4 weeks after axitinib initiation compared with those with progressive disease (*P* = .022). The median progression‐free survival (PFS) and median overall survival (OS) in patients with increased serum PAI‐1 level from pre‐treatment to 4 weeks after axitinib initiation were significantly shorter than those with decreased serum PAI‐1 level (*P* = .027 and *P* = .026, respectively). Increased serum PAI‐1 level from pre‐treatment to 4 weeks after axitinib initiation was an independent prognostic marker for shorter PFS and OS in multivariate analyses (*P* = .015 and *P* = .032, respectively). The immunohistochemical staining intensity of PAI‐1 in tumor specimens was significantly associated with Fuhrman grade and presence of distant metastasis (*P* = .026 and *P* = .010, respectively).

**Conclusions:**

The initial change in serum PAI‐1 level in the early stage of axitinib treatment could be a useful prognostic biomarker in patients with mRCC.

## 1 INTRODUCTION

In 2017, the age‐adjusted incidence and mortality rates of renal cell carcinoma (RCC) in Japanese men were 11.5 and 2.8 per 100 000 person‐years, respectively.^1^ Distant metastasis is observed in approximately 20% to 30% of patients with RCC at the time of initial diagnosis.^2^ Although current first‐line treatment for patients with metastatic RCC (mRCC) is either an immune‐checkpoint inhibitor (ICI) or vascular endothelial growth factor (VEGF)‐directed multitargeted tyrosine kinase inhibitors (TKIs),^3^ TKIs improved overall survival (OS) in patients with mRCC with a median value of 8.5 to 14.4 months from 2002 to 2008.^4^ Although the treatment paradigm for mRCC is currently shifting from TKIs to ICIs with or without concurrent use of TKIs, personalized biomarker‐guided sequential or combination therapies for predicting the efficacy and adverse effects of TKIs are still strongly required for patients with mRCC.^3^

For appropriate use of TKIs in individual patients, useful biomarkers which can be measured during treatment to predict treatment effect, resistance, and prognosis are strongly required. As strategies to predict the treatment effect and prognosis during treatment, serum TKI level can be measured.^5^ Pre‐treatment evaluation of genetic polymorphisms of drug‐metabolizing enzymes and transporters can predict the serum TKI level.^5^ In addition, serum VEGF‐C, sVEGFR‐2, and sVEGFR‐3 levels,^6^, ^7^, ^8^ and the number of endothelial cells in circulating blood^9^ have been reported to be biomarkers that correlate with treatment effect and prognosis. However, other potential biomarkers relevant to personalized therapy including TKIs and immunotherapies have not been investigated.

Axitinib is a TKI selective for VEGFR‐1, ‐2, and ‐3. Patients with mRCC treated with axitinib as second‐line therapy had a significantly longer progression‐free survival (PFS) than those treated with sorafenib in a randomized, multicenter phase III trial.^10^ In this study, we aimed to analyze various potentially prognostic serum cytokines involved in cancer angiogenesis and cell proliferation using the multiplex immunoassay method before treatment and 4 weeks after axitinib initiation in patients with mRCC. We comprehensively explored biomarkers which can predict the clinical effect and prognosis in patients with mRCC treated with axitinib.

## 2 MATERIAL AND METHODS

### 2.1 Patients

From September 2012 to October 2015, 44 patients with mRCC at the Akita University Hospital were enrolled. An approval (#924) was obtained by Akita University Hospital Institutional Review Board in accordance with the ethical standards based on the Declaration of Helsinki and its later amendments. Written informed consent was obtained by all the patients who participated in this study. Serum samples were obtained before treatment and 4 weeks after axitinib initiation. Patient characteristics are presented in Table 1. The International Metastatic Renal Cell Carcinoma Database Consortium (IMDC) risk classification at the axitinib initiation treatment was favorable in 11 (25.0%), intermediate in 30 (68.2%), and poor in 7 (15.9%). Twenty‐six (59.1%) patients received no other therapies before axitinib. Axitinib treatment was initiated at 10 mg/day twice daily; thereafter, the dosage was increased or decreased according to the discretion of the attending physician based on serum axitinib level, adverse events, and treatment effect. Evaluation of the therapeutic effect was based on the Response Evaluation Criteria in Solid Tumors v1.1.

**Table 1:** Patients characteristics of the 44 patients with metastatic renal cell carcinoma treated with axitinib

		No. of patients (%) *n* = 44
Gender	Male	31 (70.5)
	Female	13 (29.5)
Age	Median [range]	66.5 [24‐83]
BMI	Median [range]	22.7 [16.1‐31.8]
IMDC risk group classification	Favorable	8 (18.2)
	Intermediate	24 (54.5)
	Poor	7 (15.9)
	Not available	5 (11.4)
Histological type	Clear cell	36 (81.8)
	Chromophobe	2 (4.5)
	Xp translocation	4 (9.1)
	Sarcomatoid	2 (4.5)
Nephrectomy	Yes	41 (93.2)
	No	3 (6.8)
Target organ	Lung	29 (65.9)
	Lymph node	14 (31.8)
	Bone	11 (25.0)
	Liver	5 (11.4)
Previous treatment	Yes	18 (40.9)
	At least one previous molecular‐targeted agent	12 (66.7)
	Sunitinib	11 (61.1)
	Sorafenib	4 (22.2)
	Everolimus	7 (38.9)
	Temsirolimus	1 (5.6)
	Cytokines only	6 (33.3)
	No	26 (59.1)

### 2.2 Quantitative analysis of serum biomarkers

Serum samples were centrifuged at 3000 revolutions per min for 10 minutes, and stored at −80°C prior to analysis. Beads array analysis using the Bio‐Plex Pro Cancer Biomarker assay kit1 and kit2 (Bio‐Rad, Hercules, California) was performed to measure 34 cytokines and tumor growth factors.

Briefly, the capture antibody‐coupled beads were first incubated with antigen standards, quality control samples, and serum samples in 96‐well plates, followed by incubation with biotinylated detection antibodies. Samples were diluted 1:4 using sample diluent. After washing the unbound biotinylated detection antibodies, the beads were incubated with a reporter streptavidin‐phycoerythrin (SA‐PE) conjugate. Following the removal of excess SA‐PE, the beads were passed through the 2‐laser flow cytometer Bio‐Plex array reader (Bio‐Plex 200 system, Bio‐Rad), which measures the fluorescence of the bead and the bound SA‐PE. Details of the procedure have been described previously.^11^ Assay incubations were performed at room temperature. All washes were performed using the Bio‐Plex Pro wash station. Data acquisition was performed using Bio‐Plex manager TM 6.0. Using the automatic calibration curve optimization function, the recovery rate was regressed to be in the range of approximately 70% to 130%. All samples were assayed in duplicate.

The following biomarkers were determined using the Bio‐Plex Pro Human Cancer Biomarker Panel kit1 (#171‐AC500M, Bio‐Rad): soluble epidermal growth factor receptor (sEGFR), fibroblast growth factor basic (FGF‐basic), soluble VEGF receptor (sVEGFR)‐1, sVEGFR‐2, platelet endothelial cell adhesion molecule‐1 (PECAM‐1), platelet‐derived growth factor‐AB/BB (PDGF‐AB/BB), granulocyte‐colony stimulating factor (G‐CSF), hepatocyte growth factor (HGF), tyrosine kinase sHER‐2/neu (erbB‐2), tyrosine kinase sTIE2, sIL‐6Rα, *follistatin*, prolactin (PRL), leptin, and osteopontin. In addition, the following biomarkers were determined using the Bio‐Plex Pro Human Cancer Biomarker Panel kit2 (#171‐AC600M, Bio‐Rad): VEGF‐A, VEGF‐C, VEGF‐D, epidermal growth factor receptor (EGFR), heparin‐binding epidermal growth factor‐like growth factor (HB‐EGF), *placental growth factor (*PLGF*)*, *transforming growth factor‐α (*TGF‐α*)*, tumor necrosis factor‐*α (*TNF‐α*)*, insulin‐like growth factor‐binding protein 1 (IGFBP‐1), soluble Fas ligand (sFASL), IL‐6, IL‐8, IL‐18, plasminogen activator inhibitor‐1 (PAI‐1), urokinase plasminogen activator (uPA), angiopoietin‐2, sCD40L, and endoglin.

### 2.3 Immunohistochemistry staining

Tumor specimens obtained by radical nephrectomy or biopsy were fixed in 20% formalin, embedded in paraffin, and evaluated for expression of PAI‐1. Specimens were sliced into 3 μm sections and immunohistochemically analyzed using anti‐PAI‐1 antibody (#66705, Abcam, Cambridge, UK). Peroxidase and 3,3‐diaminobenzidine (DAB) were used as labeling enzyme and chromogenic substrate, respectively. Immunohistochemistry (IHC) staining was assessed using an automated quantitative pathology imaging system workstation (Mantra, PerkinElmer, Waltham, Massachusetts). DAB‐positive cells were detected, and the staining intensity was scored using inForm ver. 2.3 software (PerkinElmer). Five representative areas were photographed with a 400‐fold field of view, and nuclei were automatically recognized. Staining intensity was measured radially from the nucleus, and DAB staining was recognized around the cell membrane (Figure S1). The positive threshold for staining intensity per cell was defined as ≥25% of the maximum staining intensity. The percentage of cells exceeding the threshold was counted, and the average value of the five visualized areas was scored as the final IHC staining intensity.

### 2.4 Statistical analysis

The Kolmogorov‐Smirnov test was used for nonparametric analysis of the serum biomarkers because of their nonnormal distribution. The relationships between serum biomarker level, treatment response, IHC staining intensity, and pathological parameters were evaluated using the Mann‐Whitney *U* test. Bonferroni's correction was applied in the multiple comparison. Fisher's exact test was used to examine the proportion of patients between groups. The Kaplan‐Meier method was used to plot time‐to‐event curves, and statistical significance was estimated using the log‐rank test. The Cox proportional hazard model was used to determine independent prognostic factors of PFS and OS. *P* < .05 was considered as statistically significant. All statistical analyses were performed using SPSS statistics version 23 (IBM, New York).

## 3 RESULTS

### 3.1 Change in serum biomarker levels from pre‐treatment to 4 weeks after axitinib initiation

Among the 34 measured cancer‐related biomarkers, the median serum level of sTIE2, sVEGFR‐1, sVEGFR‐2, and Ang2 significantly decreased from pre‐treatment to 4 weeks after axitinib initiation (*P* < .001, *P* = .036, *P* < .001, and *P* = .006, respectively; Table 2).

**Table 2:** List of the determined biomarkers and their serum level of pre‐treatment and 4 weeks after initiation of axitinib

Protein name	Abbreviations	Pre‐treatment		4 weeks after initiation of axitinib		*P* value	Number of patients for change in the serum level	
		Median (pg/mL)	Range	Median (pg/mL)	Range		Increased (*n*)	Decreased (*n*)
Bio‐Plex Pro Human Cancer Biomarker Panel kit1								
Soluble epidermal growth factor receptor	sEGFR	14 779	12 669‐18 295	15 200	13 386‐20 398	.032	29	15
Fibroblast growth factor basic	FGF‐basic	194	161‐218	183	160‐215	.090	17	27
Follistatin	Follistatin	707	506‐948	629	497‐1279	.375	23	21
Granulocyte‐colony stimulating factor	G‐CSF	82	60‐93	76	62‐90	.255	19	25
Tyrosine kinase soluble HER‐2/neu	erbB‐2	2186	1705‐3348	2754	1618‐3288	.273	24	20
Hepatocyte growth factor	HGF	1246	1022‐2783	1305	1050‐3174	.666	23	21
Soluble IL‐6Rα	sIL‐6Rα	10 180	8227‐11 940	10 507	8329‐12 732	.161	28	16
Leptin	Leptin	1907	1016‐4364	2134	924‐3545	.788	21	23
Osteopontin	OPN	70 999	45 785‐90 563	69 869	47 384‐94 053	.972	22	22
Platelet‐derived growth factor‐AB/BB	PDGF‐AB/BB	2732	1941‐4126	2796	1939‐3815	.735	22	22
Platelet endothelial cell adhesion molecule‐1	PECAM‐1	2981	2539‐4093	3257	2662‐3849	.926	26	18
Prolactin	PRL	6029	4378‐11 048	8036	5323‐17 673	.010	33	11
Stem cell factor	SCF	219	197‐267	219	193‐247	.076	16	28
Tyrosine kinase soluble TIE2	sTIE‐2	6168	5137‐8635	5510	4082‐7099	<.001	9	35
Soluble vascular endothelial growth factor receptor‐1	sVEGFR‐1	219	138‐304	188	140‐257	.036	17	27
Soluble vascular endothelial growth factor receptor‐1	sVEGFR‐2	3558	2728‐4098	2830	2209‐3217	<.001	7	37
Bio‐Plex Pro Human Cancer Biomarker Panel kit2								
Angiopoietin‐2	Ang2	954	567‐1306	751	292‐1366	.006	13	31
Soluble CD40 ligand	sCD40L	412	286‐487	390	308‐495	.797	22	22
Epidermal growth factor receptor	EGF	58	29‐89	62	33‐99	.161	28	16
Endoglin	ENG	906	459‐1197	817	413‐1186	.138	19	25
Soluble Fas ligand	sFASL	298	259‐396	278	226‐420	.118	15	29
Heparin‐binding epidermal growth factor‐like growth factor	HB‐EGF	79	54‐96	71	46‐102	.197	21	23
Insulin‐like growth factor‐binding protein 1	IGFBP‐1	12 372	4731‐18 729	11 605	3447‐28 333	.135	26	18
Interleukin‐6	IL‐6	80	33‐102	68	26‐108	.930	23	21
Interleukin‐8	IL‐8	24	13‐29	24	12‐34	.718	23	21
Interleukin‐18	IL‐18	135	105‐182	160	91‐207	.243	23	21
Plasminogen activator inhibitor‐1	PAI‐1	110 156	74 073‐165 898	107 590	76 894‐147 861	.991	24	20
Placental growth factor	PLGF	86	43‐128	102	52‐141	.067	30	14
Transforming growth factor‐α	TGF‐α	60	46‐81	52	38‐86	.700	21	23
Tumor necrosis factor‐α	TNF‐α	44	16‐67	39	14‐61	.280	20	24
Urokinase plasminogen activator	uPA	228	74‐340	210	69‐371	.981	21	23
Soluble vascular endothelial growth factor A	VEGF‐A	580	459‐754	610	382‐862	.401	25	19
Soluble vascular endothelial growth factor C	VEGF‐C	959	671‐1075	921	580‐1167	.815	24	20
Soluble vascular endothelial growth factor D	VEGF‐D	862	498‐1633	753	466‐1600	.155	19	25

In contrast, the median serum level of sEGFR and PRL significantly increased from pre‐treatment to 4 weeks after axitinib initiation (*P* = .032 and *P* = .010, respectively; Table 2). Using Bonferroni's correction, only sTIE2, sVEGFR‐2, and PRL were significantly decreased or increased. The number of patients for each serum biomarker who exhibited a decrease or increase in level is shown in Table 2.

### 3.2 Relationship between serum biomarker levels and treatment response

The treatment responses of 42 patients treated with axitinib were partial remission (PR) in 16 (38.1%) patients, stable disease (SD) in 20 (47.6%), and progressive disease (PD) in 6 (14.3%). Two patients were excluded because of unknown response. The median serum PDGF‐AB/BB and sVEGFR‐2 levels at baseline were significantly higher in the six patients with PD than in the 36 patients with PR or SD (*P* = .040 and *P* = .003, respectively); however, the baseline median serum PAI‐1 level was significantly lower in the patients with PD than those with PR or SD (*P* = .048) (Table S1). Using Bonferroni's correction, there was no significant relationship.

The proportion of patients with decreased serum level of PAI‐1 and IL‐18 from pre‐treatment to 4 weeks after axitinib initiation was significantly higher in patients with PR or SD compared to those with PD (*P* = .022 and *P* = .022, respectively; Table S2). The proportion of patients with decreased serum levels of endoglin, IL‐6, and VEGF‐A from pre‐treatment to 4 weeks after axitinib initiation was significantly higher in patients with PR than those with SD or PD (*P* = .011, *P* = .025, and *P* = .029, respectively; Table S2). Using Bonferroni's correction, there was no significant relationship.

### 3.3 Relationship between serum biomarker levels and PFS and OS

The presence of lymph node swelling on initial imaging studies (cN1) and baseline serum leptin level lower than the median were independent factors related to worse PFS in multivariate analysis (*P* < .001 and *P* = .026; Table S3). No independent factor related to OS was found using baseline serum biomarker level (Table S4).

**Figure 1:**
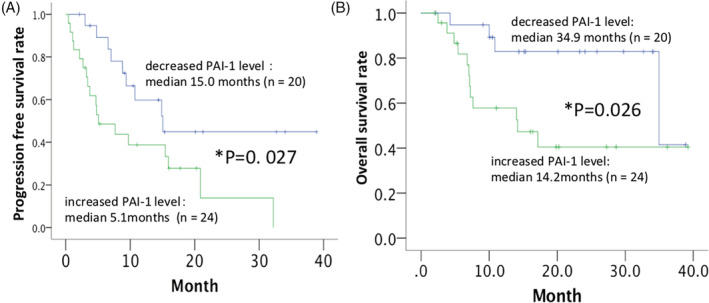
Kaplan‐Meier curves comparing, A, progression‐free survival, and B, overall survival in patients with decreased or increased serum plasminogen activator inhibitor‐1 (PAI‐1) level from pre‐treatment to 4 weeks after axitinib initiation

Patients with increased serum PAI‐1 level from pre‐treatment to 4 weeks after axitinib initiation had significantly shorter PFS and OS than those with decreased serum PAI‐1 (15.0 months vs 5.1 months, *P* = .027 and 34.9 months vs 14.2 months, *P* = .026, respectively; Figure 1A,B). The presence of lymph node swelling on initial imaging studies (cN1) and increased serum PAI‐1 level from pre‐treatment to 4 weeks after axitinib initiation were independent prognostic factors for shorter PFS (*P* < .001 and *P* = .015, respectively; Table 3). Increased serum PAI‐1 level from pre‐treatment to 4 weeks after axitinib initiation was also an independent prognostic marker for shorter OS (*P* = .032; Table 4).

**Table 3:** Cox proportional hazard model to predict the shorter progression‐free survival using baseline clinical parameter and change in the serum biomarker level from pre‐treatment to 4 weeks after initiation of axitinib

Variable	Univariate analysis			Multivariate analysis (stepwise)		
	HR	95% CI	*P* value	HR	95%CI	*P* value
Age (<median vs >median)	0.747	0.346‐1.611	.456			
Gender (male vs female)	1.048	0.441‐2.493	.915			
BMI (<25 vs ≧25)	0.788	0.359‐1.730	.553			
Previous treatment (no vs yes)	0.850	0.349‐1.831	.678			
pT (≧pT2 vs pT1)	1.508	0.627‐3.628	.359			
cN (≧cN1 vs cN0)	5.476	2.039‐14.704	.001	10.616	3.287‐34.280	<.001
LVI (yes vs no)	1.226	0.409‐3.672	.716			
Grade (G2‐3 vs G1)	1.141	0.586‐2.219	.699			
Number of metastasis (≧3 vs 0‐2)	1.937	0.838‐4.477	.122			
Lung metastasis (yes vs no)	1.019	0.441‐2.353	.965			
Liver metastasis (yes vs no)	3.236	1.180‐8.875	.022	2.854	0.843‐9.662	.092
Bone metastasis (yes vs no)	1.890	0.823‐4.338	.133			
CRP (≧ULN vs <ULN)	1.114	0.486‐2.554	.798			
Alb (<LLN vs >LLN)	2.630	0.991‐6.981	.052			
Hb (<LLN vs >LLN)	1.859	0.858‐4.028	.112			
Thrombocyte(<ULN vs ≧ULN)	1.802	0.674‐4.819	.241			
sEGFR (increased vs decreased)	0.787	0.348‐1.780	.565			
FGF‐basic (increased vs decreased)	1.217	0.686‐2.158	.501			
Follistatin (increased vs decreased)	0.859	0.396‐1.863	.700			
G‐CSF (increased vs decreased)	1.124	0.525‐2.406	.763			
erbB‐2 (increased vs decreased)	1.039	0.471‐2.291	.925			
HGF (increased vs decreased)	1.492	0.689‐3.230	.310			
IL‐6Rα (increased vs decreased)	1.573	0.687‐3.605	.284			
Leptin (increased vs decreased)	0.953	0.446‐2.036	.900			
OPN (increased vs decreased)	1.078	0.503‐2.313	.847			
PDGF‐AB/BB (increased vs decreased)	0.860	0.402‐1.837	.697			
PECAM‐1 (increased vs decreased)	1.377	0.611‐3.104	.441			
PRL (increased vs decreased)	1.233	0.519‐2.929	.635			
SCF(increased vs decreased)	1.002	0.458‐2.193	.996			
TIE2 (increased vs decreased)	0.711	0.283‐1.782	.466			
sVEGFR‐1 (increased vs decreased)	0.764	0.378‐1.541	.451			
sVEGFR‐2 (increased vs decreased)	0.839	0.313‐2.245	.726			
Ang2 (increased vs decreased)	0.809	0.341‐1.921	.631			
sCD40L (increased vs decreased)	2.135	0.956‐4.770	.064			
EGF (increased vs decreased)	1.809	0.763‐4.289	.178			
ENG (increased vs decreased)	1.667	0.780‐3.563	.188			
sFASL (increased vs decreased)	1.457	0.665‐3.193	.347			
HB‐EGF (increased vs decreased)	2.233	1.027‐4.854	.043	1.937	0.208‐60.373	.561
IGFBP‐1 (increased vs decreased)	1.359	0.619‐2.986	.444			
IL‐6 (increased vs decreased)	2.328	1.053‐5.143	.037	1.037	0.332‐3.237	.949
IL‐8 (increased vs decreased)	1.935	0.879‐4.258	.101			
IL‐18 (increased vs decreased)	1.675	0.759‐3.694	.201			
PAI‐1 (increased vs decreased)	2.412	1.075‐5.412	.027	3.896	1.306‐11.623	.015
PLGF (increased vs decreased)	2.671	1.008‐7.075	.048	2.018	0.547‐8.127	.279
TGF‐α (increased vs decreased)	2.485	1.114‐5.546	.026	0.912	0.089‐9.039	.938
TNF‐α (increased vs decreased)	1.995	0.928‐4.291	.077			
uPA (increased vs decreased)	1.444	0.693‐3.008	.327			
VEGF‐A (increased vs decreased)	1.435	0.656‐3.142	.366			
VEGF‐C (increased vs decreased)	1.924	0.875‐4.233	.104			
VEGF‐D (increased vs decreased)	1.608	0.753‐3.432	.220			

**Table 4:** Cox proportional hazard model to predict the shorter overall survival using baseline clinical parameter and change in the serum biomarker level from pre‐treatment to 4 weeks after initiation of axitinib

Variable	Univariate			Multivariate		
	HR	95% CI	*P* value	HR	95%CI	*P* value
Age (<median vs >median)	0.480	0.174‐1.324	.156			
Gender (male vs female)	0.854	0.274‐2.658	.785			
BMI (<25 vs ≧25)	0.602	0.208‐1.745	.350			
Previous treatment (no vs yes)	0.534	0.182‐1.568	.253			
pT (≧pT2 vs pT1)	1.233	0.386‐3.942	.724			
cN (≧cN1 vs cN0)	4.691	1.562‐14.089	.006	2.292	0.483‐10.883	.297
LVI (yes vs no)	1.494	0.326‐6.853	.606			
Grade (G2‐3 vs G1)	1.439	0.597‐3.473	.418			
Number of metastasis (≧3 vs 0‐2)	4.104	1.487‐11.321	.006	2.709	0.357‐20.533	.335
Lung metastasis (yes vs no)	0.912	0.311‐2.674	.867			
Liver metastasis (yes vs no)	2.841	0.904‐8.924	.074			
Bone metastasis (yes vs no)	3.255	1.198‐8.846	.021	2.472	0.370‐16.492	.35
CRP (≧ULN vs <ULN)	3.102	0.703‐13.684	.135			
Alb (<LLN vs >LLN)	3.417	0.769‐15.175	.106			
Hb (<LLN vs >LLN)	3.382	1.090‐10.496	.035	1.996	0.534‐7.453	.304
Thrombocyte(<ULN vs ≧ULN)	3.046	0.957‐9.699	.059			
sEGFR (increased vs decreased)	0.753	0.273‐2.079	.584			
FGF‐basic (increased vs decreased)	1.119	0.508‐2.464	.781			
Follistatin (increased vs decreased)	0.969	0.363‐2.586	.949			
G‐CSF (increased vs decreased)	0.622	0.215‐1.799	.381			
erbB‐2 (increased vs decreased)	0.701	0.261‐1.880	.481			
HGF (increased vs decreased)	1.753	0.637‐4.824	.277			
IL‐6Rα (increased vs decreased)	2.130	0.684‐6.637	.192			
Leptin (increased vs decreased)	1.203	0.451‐3.210	.712			
OPN (increased vs decreased)	1.498	0.533‐4.212	.443			
PDGF‐AB/BB (increased vs decreased)	0.678	0.245‐1.874	.454			
PECAM‐1 (increased vs decreased)	0.906	0.336‐2.443	.846			
PRL (increased vs decreased)	0.846	0.294‐2.437	.757			
SCF(increased vs decreased)	1.729	0.647‐4.625	.275			
TIE2 (increased vs decreased)	0.651	0.185‐2.289	.503			
sVEGFR‐1 (increased vs decreased)	0.634	0.244‐1.647	.349			
sVEGFR‐2 (increased vs decreased)	1.104	0.286‐3.598	.983			
Ang2 (increased vs decreased)	1.279	0.455‐3.595	.641			
sCD40L (increased vs decreased)	1.173	0.434‐3.173	.753			
EGF (increased vs decreased)	0.804	0.292‐2.219	.674			
ENG (increased vs decreased)	1.175	0.441‐3.133	.747			
sFASL (increased vs decreased)	1.228	0.443‐3.399	.693			
HB‐EGF (increased vs decreased)	1.486	0.549‐4.025	.436			
IGFBP‐1 (increased vs decreased)	1.237	0.449‐3.408	.680			
IL‐6 (increased vs decreased)	2.349	0.813‐6.783	.115			
IL‐8 (increased vs decreased)	0.916	0.331‐2.531	.865			
IL‐18 (increased vs decreased)	1.539	0.559‐4.240	.404			
PAI‐1 (increased vs decreased)	3.376	1.086‐10.497	.036	5.316	1.154‐24.488	.032
PLGF (increased vs decreased)	1.424	0.453‐4.474	.545			
TGF‐α (increased vs decreased)	1.486	0.549–4.025	.436			
TNF‐α (increased vs decreased)	1.130	0.424‐3.015	.807			
uPA (increased vs decreased)	2.240	0.819‐6.123	.116			
VEGF‐A (increased vs decreased)	1.057	0.383‐2.918	.915			
VEGF‐C (increased vs decreased)	1.508	0.547‐4.152	.427			
VEGF‐D (increased vs decreased)	0.846	0.312‐2.298	.743			

### 3.4 Relationship between IHC staining intensity and clinical parameters

Of the 44 patients enrolled in this study, 41 (93.2%) underwent radical nephrectomy and 3 (6.8%) underwent tumor biopsy. IHC analysis using PAI‐1 antibody was available in 39 specimens from 36 nephrectomies and 3 biopsies. The median IHC staining intensity of PAI‐1 was significantly higher in patients with metastatic disease at the time of diagnosis than those with nonmetastatic disease (*P* = .010; Table 5), as well as in patients with Fuhrman grade ≥ 3 tumors than in those with grade ≤ 2 (*P* = .026; Table 5). There was no significant relationship between PAI‐1 staining intensity and PFS or OS (Figure S2), and between PAI‐1 staining intensity and serum baseline PAI‐1 level (*r* ^2^ = 0.053, *ρ* = −0.02, *P* = .904).

**Table 5:** Relationship between IHC staining intensity of PAI‐1 and pathological parameters of patients treated with axitinib

*n* = 39	pT				*P* value	Metastasis				*P* value	Fuhrman grade				*P* value
	≤pT2 (*n* = 19)		≥pT3 (*n* = 20)			M0 (*n* = 17)		M1 (*n* = 22)			≤G2 (*n* = 12)		≥G3 (*n* = 26)		
	Median	Range	Median	Range		Median	Range	Median	Range		Median	Range	Median	Range	
IHC score (median)	0.686	0.268‐0.857	0.668	0.437‐0.745	.955	0.289	0.147‐0.724	0.738	0.600‐0.821	.010	0.281	0.083‐0.726	0.728	0.604‐0.788	.026

## 4 DISCUSSION

The multiplex immunoassay method is a beads array in which various antibodies are loaded on the beads measured by flow cytometry. Previous reports have comprehensively measured angiogenic factors using serum samples from patients with colorectal, ovarian and small cell lung cancer^12^, ^13^, ^14^ and urine samples from patients with bladder cancer.^15^, ^16^ However, few studies have explored biomarkers as predictive factors in patients with metastatic disease using multiplex immunoassay techniques. Although we expected biomarkers other than sVEGFRs to show predictive value in this study, serum PAI‐1 level was the only biomarker associated with therapeutic effect, PFS, and OS after axitinib treatment in patients with mRCC.

PAI‐1 usually exists in vascular endothelial cells, liver, platelets, and adipocytes, and functions as the principal inhibitor of urokinase‐type plasminogen activator (uPA) and its receptor (uPAR) system in fibrinolysis. Furthermore, ≥90% of PAI‐1 is contained in platelets and released into the bloodstream under conditions of vascular endothelial injury.^17^ The uPA‐uPAR complex activates matrix metalloprotease (MMP) and promotes cancer invasion. Since PAI‐1 forms a PAI‐1‐uPA‐uPAR complex and acts repressively on uPA‐uPAR, PAI‐1 is expected to have a tumor‐suppressive effect. However, tumor PAI‐1 expression has been reportedly associated with tumor progression.^18^, ^19^ This paradox has been explained by rapid internalization of the PAI‐1‐uPA‐uPAR complex by low‐density lipoprotein receptor‐related protein.

Regarding the relationship between tumor PAI‐1 expression and RCC prognosis, IHC staining intensity of cytoplasmic PAI‐1 in paraffin specimens has been previously associated with shorter disease‐free survival, OS, and cause‐specific survival (CSS) in patients with RCC.^20^, ^21^, ^22^, ^23^, ^24^, ^25^ In addition, high tissue level of PAI‐1 in fresh‐frozen RCC specimens measured using enzyme‐linked immunosorbent assay has been associated with high grade tumors^26^ and shorter CSS.^27^ In this study, PAI‐1 staining intensity was associated with the presence of metastasis at the time of diagnosis and histologic Fuhrman grade, but not with PFS and OS. However, this study evaluated staining intensity using an automated quantitative imaging system but not using microscopic manual examination as in previous studies. Further IHC studies using an automated quantitative imaging system with larger numbers of patients are required.

In this study, decreased serum PAI‐1 level after axitinib treatment was related to improved treatment effect and prognosis. However, the serum PAI‐1 level at baseline was not related to the axitinib effect or prognosis. Significant decreases have been observed in both serum PAI‐1 and VEGF levels after treatment in a previous study of sunitinib plus interferon in patients with mRCC,^28^ whereas no significant decrease in serum PAI‐1 level after treatment was observed in our axitinib study. In breast cancer, lower pre‐treatment plasma PAI‐1 level was an independent prognostic factor for PFS and OS,^29^ and plasma PAI‐1 level did not correlate with PAI‐1 immunostaining intensity.^30^ Our results with an inverse correlation between plasma levels and immunostaining intensity were similar to those in the breast cancer results. Since the serum PAI‐1 level would reflect PAI‐1 released from the tumor, endothelium, and platelets, the successful suppression of both tumor and systemic angiogenesis by axitinib might decrease the serum PAI‐1 level. The decrease of the serum PAI‐1 level might reflect the change of the tumor microenvironment induced by axitinib which could be associated with the better prognosis. It is assumed that PAI‐1 expressed in tumor cells and released into circulation may have a different biological role in patients with mRCC. Although an *in vivo* murine study using systemic administration of the PAI‐1 inhibitor SK‐216 for lung cancer and melanoma indicated that PAI‐1 generated by host rather than tumor cells plays a determinant role in the anticancer effect,^31^ further accumulation of biomarker data in patients with mRCC treated with axitinib is warranted to verify the results.

Additionally, the median serum level of sVEGFR‐1 and sVEGFR‐2 decreased significantly from pre‐treatment to 4 weeks after axitinib initiation, and the decline of serum sVEGFR‐2 level was associated with treatment response in this study. However, sVEGFRs were not independent predictive factors for PFS or OS using baseline serum biomarker level or change in level after treatment. These results are partially consistent with previous studies that reported sVEGFR‐2 and sVEGFR‐3 levels were significant prognostic factors after sunitinib treatment in patients with mRCC.^6^, ^7^ Although serum PAI‐1 and sVEGFRs have been identified as markers of tumor hypoxia, and might be affected by systemic VEGF‐directed inhibitors,^28^, ^32^ serum PAI‐1 level may be a more useful prognostic biomarker than serum sVEGFRs in this axitinib study.

There are several important limitations of this study. First, PAI‐1 is ideally measured in plasma, however we used serum samples in this study, which might affect the results. Second, the PAI‐1 level measured in this study was not pure PAI‐1 but a complex in the blood. The antibody on the beads of the Bio‐Plex Pro Human Cancer Biomarker Panel 2 in this study is an anti‐total PAI‐1 antibody, which measures the sum of the active type, latent type, vitronectin complex, tissue‐type plasminogen activator complex, and uPA complex. Third, 40% of patients received multiple therapies prior to axitinib treatment, which might affect the interpretation of the results. To verify our results, future studies measuring plasma PAI‐1 level in larger RCC cohorts should be conducted.

## 5 CONCLUSIONS

The initial changes in serum PAI‐1 level at the early stage of axitinib treatment could be a useful prognostic biomarker in patients with mRCC.

## FUNDING

This work was supported by the grant numbers 25293332, 16H02679, and 23590168 from the Japanese Society for the Promotion of Science and AMED‐CREST, Japan Agency for Medical Research and Development (AMED).

## CONFLICT OF INTEREST

The authors declare no conflicts of interest.

## AUTHOR CONTRIBUTIONS

Conceptualization: Naoko Honma, Takamitsu Inoue, and Norihiko Tsuchiya

Data curation: Naoko Honma, Takamitsu Inoue, and Norihiko Tsuchiya

Formal analysis: Naoko Honma and Takamitsu Inoue

Funding acquisition: Takamitsu Inoue, Norihiko Tsuchiya, and Tomonori Habuchi

Investigation: Naoko Honma, Mingguo Huang, and Takamitsu Inoue

Methodology: Naoko Honma, Takamitsu Inoue, and Norihiko Tsuchiya

Project administration: Naoko Honma and Takamitsu Inoue

Resources: Naoko Honma, Takamitsu Inoue, Atsushi Koizumi, Ryohei Yamamoto, Taketoshi Nara, Sohei Kanda, Mitsuru Saito, Kazuyuki Numakura, and Shintaro Narita

Software: Naoko Honma, Mingguo Huang, and Takamitsu Inoue

Supervision: Shintaro Narita, Shigeru Satoh, and Tomonori Habuchi

Validation: Shintaro Narita

Visualization: Naoko Honma and Takamitsu Inoue

Writing ‐ original draft preparation: Naoko Honma

Writing ‐ review and editing: Takamitsu Inoue, Shintaro Narita, Shigeru Satoh, and Tomonori Habuchi

 All authors have read and approved the final version of the manuscript.

## TRANSPARENCY STATEMENT

The corresponding author, Takamitsu Inoue, affirms that this manuscript is an honest, accurate, and transparent account of the study being reported; that no important aspects of the study have been omitted; and that any discrepancies from the study as planned (and, if relevant, registered) have been explained.

## Supporting information


**Figure S1**. Representative PAI‐1‐stained immunohistochemistry images. Immunohistochemistry staining was assessed using an automated quantitative pathology imaging system (Mantra, PerkinElmer). DAB‐positive cells were assessed, and the IHC staining intensity of PAI‐1 was scored using inForm software ver. 2.3. The IHC staining intensity of PAI‐1 was (A, D) 26.8%, (B, E) 53.6%, and (C, F) 90.4%. The yellow and blue nucleus indicated the PAI‐1 positive and negative on the tumor cell membrane, respectively (D‐F).

**Figure S2**. Kaplan‐Meier curve comparing (a) progression‐free survival and (b) overall survival in patients with >median PAI‐1 staining intensity (n = 19) or < median (n = 20), in whom the pathological specimen was available.

**Table S1**. Relationship between baseline serum biomarker level and objective responses.

**Table S2**. Relationship between change in the serum biomarker level from pre‐treatment to 4 weeks after initiation of axitinib and objective responses.

**Table S3**. Cox proportional hazard model to predict the shorter progression free survival using baseline clinical parameter and serum biomarker level.

**Table S4**. Cox proportional hazard model to predict the shorter overall survival using baseline clinical parameter and serum biomarker level.

## Data Availability

The data that support the findings of this study are openly available in “figshare” at https://figshare.com/s/ea7a0931565d9b36f1e2, DOI: 10.6084/m9.figshare.12049560.
