# Supplementary figures and images for: Prognostic value of plasminogen activator inhibitor‐1 in biomarker exploration using multiplex immunoassay in patients with metastatic renal cell carcinoma treated with axitinib
Source: Health Sci Rep. 2020 Oct 15;3(4):e197. doi: 10.1002/hsr2.197 (PMC7559632; doi:10.1002/hsr2.197)

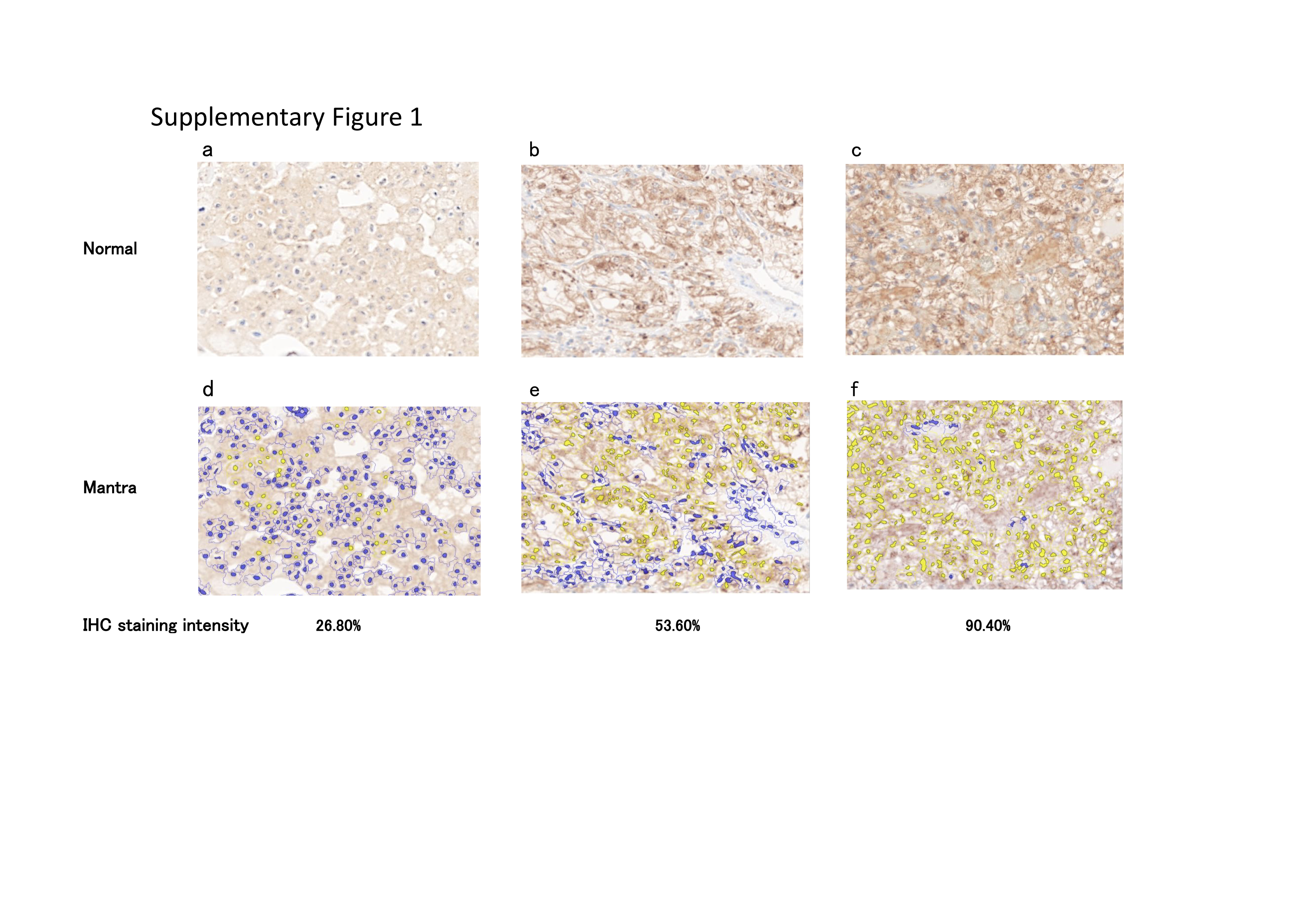

Supplement: Supplementary file 1 — Figure S1. Representative PAI‐1‐stained immunohistochemistry images. Immunohistochemistry staining was assessed using an automated quantitative pathology imaging system (Mantra, PerkinElmer). DAB‐positive cells were assessed, and the IHC staining intensity of PAI‐1 was scored using inForm software ver. 2.3. The IHC staining intensity of PAI‐1 was (A, D) 26.8%, (B, E) 53.6%, and (C, F) 90.4%. The yellow and blue nucleus indicated the PAI‐1 positive and negative on the tumor cell membrane, respectively (D‐F). [file HSR2-3-e197-s001.tiff]

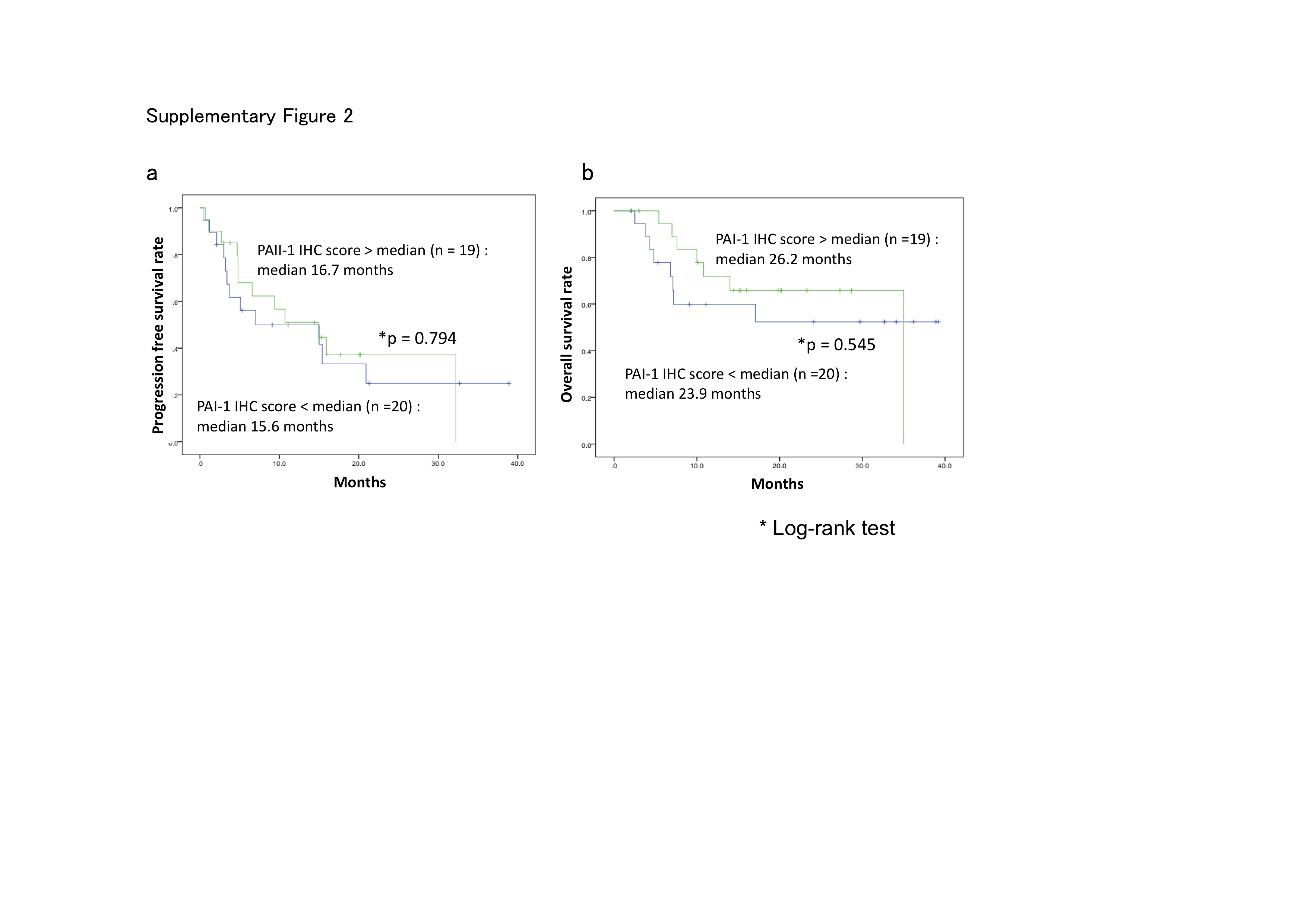

Supplement: Supplementary file 2 — Figure S2. Kaplan‐Meier curve comparing (a) progression‐free survival and (b) overall survival in patients with >median PAI‐1 staining intensity (n = 19) or < median (n = 20), in whom the pathological specimen was available. [file HSR2-3-e197-s002.tiff]
