# Supplementary material for: Prognostic value of plasminogen activator inhibitor‐1 in biomarker exploration using multiplex immunoassay in patients with metastatic renal cell carcinoma treated with axitinib
Source: Health Sci Rep. 2020 Oct 15;3(4):e197. doi: 10.1002/hsr2.197 (PMC7559632; doi:10.1002/hsr2.197)
Supplement: Supplementary file 3 — Table S1. Relationship between baseline serum biomarker level and objective responses. [file HSR2-3-e197-s003.docx]

| Supplementary Table 1: Relationship between baseline serum biomarker level and objective responses | | | |  |  |  |  |  |  |  |  |  |  |  |  |
| --- | --- | --- | --- | --- | --- | --- | --- | --- | --- | --- | --- | --- | --- | --- | --- |
|  |  |  |  |  |  |  |  |  |  |  |  |  |  |  |  |
|  | Protein Name | Abbreviations | PR (n = 16) | |  | SD+PD (n = 26) | | P value |  | PR+SD (n = 36) | |  | PD (n = 6) | | P value |
|  |  |  | median (pg/ml) | range (pg/ml) |  | median (pg/ml) | range (pg/ml) |  |  | median (pg/ml) | range (pg/ml) |  | median (pg/ml) | range (pg/ml) |  |
|  |  |  |  |  |  |  |  |  |  |  |  |  |  |  |  |
|  |  |  |  |  |  |  |  |  |  |  |  |  |  |  |  |
| Bio-Plex Pro^TM^ Human Cancer Biomarker Panel 1 | |  |  |  |  |  |  |  |  |  |  |  |  |  |  |
|  | Soluble epidermal growth factor receptor | sEGFR | 15140 | 12586-20579 |  | 14779 | 12915-18170 | 0.877 |  | 14928 | 12864-18466 |  | 15588 | 12847-21148 | 0.766 |
|  | Fibroblast growth factor basic | FGF-basic | 197 | 159-197 |  | 194 | 167-220 | 0.766 |  | 194 | 159-218 |  | 202 | 186-241 | 0.182 |
|  | Follistatin | Follistatin | 817 | 571-903 |  | 593 | 473-946 | 0.379 |  | 684 | 480-948 |  | 648 | 534-758 | 0.793 |
|  | Granulocyte-colony stimulating factor | G-CSF | 80 | 56-89 |  | 84 | 67-98 | 0.170 |  | 82 | 59-92 |  | 88 | 76-98 | 0.281 |
|  | Tyrosine kinase soluble HER-2/neu | erbB-2 | 2264 | 1761-3500 |  | 2202 | 1489-3333 | 0.979 |  | 2186 | 1590-3372 |  | 2330 | 1877-3984 | 0.636 |
|  | Hepatocyte growth factor | HGF | 1246 | 1040-3370 |  | 1233 | 1013-2229 | 0.660 |  | 1324 | 1035-2880 |  | 1081 | 1006-1669 | 0.388 |
|  | Soluble IL-6Rα | sIL-6Rα | 10341 | 9207-12159 |  | 9617 | 7798-11617 | 0.325 |  | 10180 | 8003-11824 |  | 10156 | 7967-13071 | 0.766 |
|  | Leptin | Leptin | 2161 | 1159-5062 |  | 1705 | 1016-3794 | 0.484 |  | 2077 | 1037-4364 |  | 1437 | 635-6822 | 0.562 |
|  | Osteopontin | OPN | 63494 | 45785-76345 |  | 74912 | 41055-92503 | 0.365 |  | 66418 | 42516-83478 |  | 91911 | 50820-131942 | 0.129 |
|  | Platelet-derived growth factor-AB/BB | PDGF-AB/BB | 2775 | 1978-4552 |  | 2619 | 1915-3943 | 0.856 |  | 2599 | 1882-3643 |  | 4979 | 3494-6566 | 0.003 |
|  | Platelet endothelial cell adhesion molecule -1 | PECAM-1 | 3234 | 2630-4220 |  | 2927 | 2546-4124 | 0.623 |  | 2981 | 2539-4006 |  | 3885 | 2845-4454 | 0.208 |
|  | Prolactin | PRL | 7137 | 4931-10428 |  | 5018 | 4147-11292 | 0.379 |  | 5306 | 4240-11048 |  | 6409 | 5588-9628 | 0.449 |
|  | Stem cell factor | SCF | 215 | 196-267 |  | 219 | 206-265 | 0.736 |  | 220 | 197-268 |  | 216 | 209-233 | 0.687 |
|  | Tyrosine kinase soluble TIE2 | sTIE2 | 6168 | 5301-9660 |  | 5974 | 4935-7582 | 0.453 |  | 6060 | 4960-9342 |  | 6696 | 5662-7592 | 0.611 |
|  | Soluble vascular endothelial growth factor receptor-1 | sVEGFR-1 | 207 | 111-246 |  | 237 | 158-309 | 0.170 |  | 215 | 135-304 |  | 255 | 159-281 | 0.661 |
|  | Soluble vascular endothelial growth factor receptor-1 | sVEGFR-2 | 3637 | 2787-4279 |  | 3558 | 2773-4089 | 0.836 |  | 3481 | 2698-4077 |  | 4061 | 3690-5216 | 0.040 |
|  |  |  |  |  |  |  |  |  |  |  |  |  |  |  |  |
| Bio-Plex Pro^TM^ Human Cancer Biomarker Panel 2 | |  |  |  |  |  |  |  |  |  |  |  |  |  |  |
|  | Angiopoietin-2 | Ang2 | 883 | 495-1270 |  | 992 | 574-1326 | 0.407 |  | 853 | 525-1296 |  | 1127 | 928-1973 | 0.111 |
|  | Soluble CD40 ligand | sCD40L | 437 | 376-488 |  | 355 | 243-474 | 0.204 |  | 412 | 299-468 |  | 350 | 157-524 | 0.687 |
|  | Epidermal growth factor receptor | EGF | 63 | 29-93 |  | 55 | 27-88 | 0.688 |  | 60 | 24-92 |  | 51 | 38-89 | 1.000 |
|  | Endoglin | ENG | 947 | 403-1213 |  | 874 | 481-1177 | 1.000 |  | 907 | 396-1152 |  | 987 | 632-1700 | 0.221 |
|  | Soluble Fas ligand | sFASL | 303 | 260-387 |  | 284 | 240-479 | 0.979 |  | 287 | 259-396 |  | 311 | 223-388 | 0.766 |
|  | Heparin binding-epidermal growth factor-like growth factor | HB-EGF | 79 | 54-87 |  | 78 | 49-128 | 0.641 |  | 75 | 51-94 |  | 90 | 72-120 | 0.235 |
|  | Insulin-like growth factor-binding protein 1 | IGFBP-1 | 9325 | 4290-17236 |  | 12372 | 5325-19110 | 0.641 |  | 13467 | 4758-17780 |  | 7868 | 2904-13133 | 0.297 |
|  | Interleukin-6 | IL-6 | 80 | 19-111 |  | 73 | 33-102 | 0.979 |  | 75 | 28-100 |  | 91 | 61-137 | 0.182 |
|  | Interleukin-8 | IL-8 | 25 | 43434 |  | 23 | 14-27 | 0.756 |  | 23 | 43463 |  | 26 | 22-33 | 0.350 |
|  | Interleukin-18 | IL-18 | 138 | 95-181 |  | 132 | 109-183 | 0.938 |  | 132 | 101-186 |  | 15 | 124-172 | 0.586 |
|  | Plasminogen activator inhibitor-1 | PAI-1 | 136525 | 76695-226560 |  | 95687 | 76289-153691 | 0.312 |  | 113643 | 80410-169652 |  | 69441 | 49193-116646 | 0.048 |
|  | Placental growth factor | PLGF | 94 | 35-160 |  | 86 | 45-116 | 0.756 |  | 86 | 39-126 |  | 86 | 53-156 | 0.493 |
|  | Transforming growth factor-α | TGF-α | 62 | 50-79 |  | 59 | 44-82 | 0.876 |  | 59 | 45-80 |  | 68 | 40-95 | 0.739 |
|  | Tumor necrosis factor-α | TNF-α | 56 | 13-81 |  | 44 | 21-64 | 0.534 |  | 46 | 13-65 |  | 52 | 27-84 | 0.332 |
|  | Urokinase plasminogen activator | uPA | 209 | 50-296 |  | 237 | 136-407 | 0.223 |  | 213 | 61-327 |  | 289 | 211-445 | 0.221 |
|  | Soluble vascular endothelial growth factor A | VEGF-A | 580 | 418-749 |  | 623 | 438-765 | 0.660 |  | 556 | 416-754 |  | 691 | 514-864 | 0.562 |
|  | Soluble vascular endothelial growth factor C | VEGF-C | 933 | 699-1018 |  | 915 | 647-1312 | 0.756 |  | 933 | 662-1102 |  | 883 | 641-1186 | 0.958 |
|  | Soluble vascular endothelial growth factor D | VEGF-D | 830 | 475-1121 |  | 911 | 506-1840 | 0.300 |  | 840 | 483-1633 |  | 1315 | 794-1751 | 0.369 |
|  |  |  |  |  |  |  |  |  |  |  |  |  |  |  |  |
| PR: partial response; SD: Stable disease; PD: progressive disease | |  |  |  |  |  |  |  |  |  |  |  |  |  |  |
|  |  |  |  |  |  |  |  |  |  |  |  |  | * Mann Whitney's U test | | |
