# Supplementary material for: Prognostic value of plasminogen activator inhibitor‐1 in biomarker exploration using multiplex immunoassay in patients with metastatic renal cell carcinoma treated with axitinib
Source: Health Sci Rep. 2020 Oct 15;3(4):e197. doi: 10.1002/hsr2.197 (PMC7559632; doi:10.1002/hsr2.197)
Supplement: Supplementary file 4 — Table S2. Relationship between change in the serum biomarker level from pre‐treatment to 4 weeks after initiation of axitinib and objective responses. [file HSR2-3-e197-s004.docx]

| Supplementary Table 2: Relationship between change in the serum biomarker level from pre-treatment to 4 weeks after initiation of axitinib and objective responses | | | | | | | | | | |
| --- | --- | --- | --- | --- | --- | --- | --- | --- | --- | --- |
|  |  |  |  |  |  |  |  |  |  |  |
|  | Protein Name | Abbreviations |  | PR | SD + PD | P value |  | PR + SD | PD | P value |
|  |  |  |  | (n = 16) | (n = 26) |  |  | (n = 36) | (n = 6) |  |
|  |  |  |  |  |  |  |  |  |  |  |
|  |  |  |  |  |  |  |  |  |  |  |
| Bio-Plex Pro^TM^ Human Cancer Biomarker Panel 1 | |  |  |  |  |  |  |  |  |  |
|  | Soluble epidermal growth factor receptor | sEGFR | increased | 11 | 17 | 1.000 |  | 25 | 3 | 0.383 |
|  |  |  | decreased | 5 | 9 |  |  | 11 | 3 |  |
|  | Fibroblast growth factor basic | FGF-basic | increased | 6 | 10 | 1.000 |  | 13 | 2 | 1.000 |
|  |  |  | decreased | 17 | 9 |  |  | 23 | 4 |  |
|  | Follistatin | Follistatin | increased | 8 | 13 | 1.000 |  | 19 | 2 | 0.663 |
|  |  |  | decreased | 8 | 13 |  |  | 17 | 4 |  |
|  | Granulocyte-colony stimulating factor | G-CSF | increased | 9 | 8 | 0.121 |  | 16 | 1 | 0.374 |
|  |  |  | decreased | 7 | 18 |  |  | 20 | 5 |  |
|  | Tyrosine kinase soluble HER-2/neu | erbB-2 | increased | 10 | 13 | 0.530 |  | 21 | 2 | 0.384 |
|  |  |  | decreased | 6 | 13 |  |  | 15 | 4 |  |
|  | Hepatocyte growth factor | HGF | increased | 7 | 14 | 0.751 |  | 18 | 3 | 1.000 |
|  |  |  | decreased | 9 | 12 |  |  | 18 | 3 |  |
|  | Soluble IL-6Rα | sIL-6Rα | increased | 8 | 18 | 0.327 |  | 22 | 4 | 1.000 |
|  |  |  | decreased | 8 | 8 |  |  | 14 | 2 |  |
|  | Leptin | Leptin | increased | 9 | 11 | 0.527 |  | 17 | 3 | 1.000 |
|  |  |  | decreased | 7 | 15 |  |  | 19 | 3 |  |
|  | Osteopontin | OPN | increased | 7 | 14 | 0.751 |  | 17 | 4 | 0.663 |
|  |  |  | decreased | 9 | 12 |  |  | 19 | 2 |  |
|  | Platelet-derived growth factor-AB/BB | PDGF-AB/BB | increased | 8 | 13 | 1.000 |  | 20 | 1 | 0.184 |
|  |  |  | decreased | 8 | 13 |  |  | 16 | 5 |  |
|  | Platelet endothelial cell adhesion molecule -1 | PECAM-1 | increased | 10 | 15 | 1.000 |  | 21 | 4 | 1.000 |
|  |  |  | decreased | 6 | 11 |  |  | 15 | 2 |  |
|  | Prolactin | PRL | increased | 10 | 21 | 0.281 |  | 26 | 5 | 1.000 |
|  |  |  | decreased | 6 | 5 |  |  | 10 | 1 |  |
|  | Stem cell factor | SCF | increased | 6 | 9 | 1.000 |  | 14 | 1 | 0.395 |
|  |  |  | decreased | 10 | 17 |  |  | 22 | 5 |  |
|  | Tyrosine kinase soluble TIE2 | sTIE2 | increased | 4 | 5 | 0.711 |  | 9 | 0 | 0.312 |
|  |  |  | decreased | 12 | 21 |  |  | 27 | 6 |  |
|  | Soluble vascular endothelial growth factor receptor-1 | sVEGFR-1 | increased | 6 | 10 | 1.000 |  | 15 | 1 | 0.380 |
|  |  |  | decreased | 10 | 16 |  |  | 21 | 5 |  |
|  | Soluble vascular endothelial growth factor receptor-2 | sVEGFR-2 | increased | 3 | 4 | 1.000 |  | 7 | 0 | 0.567 |
|  |  |  | decreased | 13 | 22 |  |  | 29 | 6 |  |
|  |  |  |  |  |  |  |  |  |  |  |
| Bio-Plex Pro^TM^ Human Cancer Biomarker Panel 1 | |  |  |  |  |  |  |  |  |  |
|  | Angiopoietin-2 | Ang2 | increased | 5 | 8 | 1.000 |  | 10 | 3 | 0.353 |
|  |  |  | decreased | 11 | 18 |  |  | 26 | 3 |  |
|  | Soluble CD40 ligand | sCD40L | increased | 5 | 17 | 0.055 |  | 18 | 4 | 0.665 |
|  |  |  | decreased | 11 | 9 |  |  | 18 | 2 |  |
|  | Epidermal growth factor receptor | EGF | increased | 10 | 18 | 0.742 |  | 24 | 4 | 1.000 |
|  |  |  | decreased | 6 | 8 |  |  | 12 | 2 |  |
|  | Endoglin | ENG | increased | 3 | 16 | 0.011 |  | 16 | 3 | 1.000 |
|  |  |  | decreased | 13 | 10 |  |  | 20 | 3 |  |
|  | Soluble Fas ligand | sFASL | increased | 3 | 12 | 0.102 |  | 11 | 4 | 0.164 |
|  |  |  | decreased | 13 | 14 |  |  | 25 | 2 |  |
|  | Heparin binding-epidermal growth factor-like growth factor | HB-EGF | increased | 5 | 16 | 0.111 |  | 17 | 4 | 0.663 |
|  |  |  | decreased | 11 | 10 |  |  | 19 | 2 |  |
|  | Insulin-like growth factor-binding protein 1 | IGFBP-1 | increased | 8 | 17 | 0.353 |  | 22 | 3 | 0.672 |
|  |  |  | decreased | 8 | 9 |  |  | 14 | 3 |  |
|  | Interleukin-6 | IL-6 | increased | 4 | 17 | 0.025 |  | 17 | 4 | 0.663 |
|  |  |  | decreased | 12 | 9 |  |  | 19 | 2 |  |
|  | Interleukin-8 | IL-8 | increased | 5 | 17 | 0.055 |  | 18 | 4 | 0.665 |
|  |  |  | decreased | 11 | 9 |  |  | 18 | 2 |  |
|  | Interleukin-18 | IL-18 | increased | 7 | 15 | 0.527 |  | 16 | 6 | 0.022 |
|  |  |  | decreased | 9 | 11 |  |  | 20 | 0 |  |
|  | Plasminogen activator inhibitor-1 | PAI-1 | increased | 5 | 17 | 0.055 |  | 16 | 6 | 0.022 |
|  |  |  | decreased | 11 | 9 |  |  | 20 | 0 |  |
|  | Placental growth factor | PLGF | increased | 10 | 19 | 0.510 |  | 24 | 5 | 0.647 |
|  |  |  | decreased | 6 | 7 |  |  | 12 | 1 |  |
|  | Transforming growth factor-α | TGF-α | increased | 5 | 16 | 0.111 |  | 17 | 4 | 0.663 |
|  |  |  | decreased | 11 | 10 |  |  | 19 | 2 |  |
|  | Tumor necrosis factor-α | TNF-α | increased | 5 | 15 | 0.121 |  | 16 | 4 | 0.400 |
|  |  |  | decreased | 11 | 11 |  |  | 20 | 2 |  |
|  | Urokinase plasminogen activator | uPA | increased | 6 | 13 | 0.530 |  | 15 | 4 | 0.384 |
|  |  |  | decreased | 10 | 13 |  |  | 21 | 2 |  |
|  | Soluble vascular endothelial growth factor A | VEGF-A | increased | 6 | 19 | 0.029 |  | 21 | 4 | 1.000 |
|  |  |  | decreased | 10 | 7 |  |  | 15 | 2 |  |
|  | Soluble vascular endothelial growth factor C | VEGF-C | increased | 7 | 16 | 0.344 |  | 18 | 5 | 0.197 |
|  |  |  | decreased | 9 | 10 |  |  | 18 | 1 |  |
|  | Soluble vascular endothelial growth factor D | VEGF-D | increased | 5 | 14 | 0.208 |  | 16 | 3 | 1.000 |
|  |  |  | decreased | 11 | 12 |  |  | 20 | 3 |  |
|  |  |  |  |  |  |  |  |  |  |  |
| PR: partial response; SD: Stable disease; PD: progressive disease | |  |  |  |  |  |  |  |  |  |
|  |  |  |  |  |  |  |  | * Fisher's exact test (two-sided test) | | |
