# Supplementary material for: Prognostic value of plasminogen activator inhibitor‐1 in biomarker exploration using multiplex immunoassay in patients with metastatic renal cell carcinoma treated with axitinib
Source: Health Sci Rep. 2020 Oct 15;3(4):e197. doi: 10.1002/hsr2.197 (PMC7559632; doi:10.1002/hsr2.197)
Supplement: Supplementary file 5 — Table S3. Cox proportional hazard model to predict the shorter progression free survival using baseline clinical parameter and serum biomarker level. [file HSR2-3-e197-s005.docx]

| Supplementary Table 3: Cox proportional hazard model to predict the shorter progression free survival using baseline clinical parameter and serum biomarker level. | | | | | | | | |
| --- | --- | --- | --- | --- | --- | --- | --- | --- |
|  |  |  |  |  |  |  |  | |
| Variable | Univariate analysis | | |  | Multivariate analysis (stepwise) | | | |
|  | HR | 95% CI | P value |  | HR | 95%CI | | P value |
|  |  |  |  |  |  |  |  | |
|  |  |  |  |  |  |  |  | |
| Age (< median vs >median) | 0.747 | 0.346-1.611 | 0.456 |  |  |  |  | |
| Gender (male vs female) | 1.048 | 0.441-2.493 | 0.915 |  |  |  |  | |
| BMI (<25 vs ≧25) | 0.788 | 0.359-1.730 | 0.553 |  |  |  |  | |
| Previous treatment (No vs Yes) | 0.850 | 0.349-1.831 | 0.678 |  |  |  |  | |
| pT (≧pT2 vs pT1) | 1.508 | 0.627-3.628 | 0.359 |  |  |  |  | |
| cN (≧cN1 vs cN0 ) | 5.476 | 2.039-14.704 | 0.001 |  | 10.616 | 3.287-34.280 | < 0.001 | |
| LVI (Yes vs No) | 1.226 | 0.409-3.672 | 0.716 |  |  |  |  | |
| Grade (G2-3 vs G1) | 1.141 | 0.586-2.219 | 0.699 |  |  |  |  | |
| Number of metastasis (≧3 vs 0−2) | 1.937 | 0.838-4.477 | 0.122 |  |  |  |  | |
| Lung metastasis (yes vs no) | 1.019 | 0.441-2.353 | 0.965 |  |  |  |  | |
| Liver metastasis (yes vs no) | 3.236 | 1.180-8.875 | 0.022 |  | 2.854 | 0.843-9.662 | 0.092 | |
| Bone metastasis (yes vs no) | 1.890 | 0.823-4.338 | 0.133 |  |  |  |  | |
| CRP (≧ ULN vs < ULN ) | 1.114 | 0.486-2.554 | 0.798 |  |  |  |  | |
| Alb (< LLN vs > LLN) | 2.630 | 0.991-6.981 | 0.052 |  |  |  |  | |
| Hb (< LLN vs > LLN) | 1.859 | 0.858-4.028 | 0.112 |  |  |  |  | |
| Thrombocyte( <ULN vs ≧ULN) | 1.802 | 0.674-4.819 | 0.241 |  |  |  |  | |
| sEGFR (0w ≧ median vs < median ) | 0.674 | 0.313-1.453 | 0.314 |  |  |  |  | |
| FGF-basic (0w ≧ median vs < median ) | 0.819 | 0.380-1.768 | 0.612 |  |  |  |  | |
| Follistatin (0w ≧ median vs < median ) | 0.926 | 0.426-2.014 | 0.847 |  |  |  |  | |
| G-CSF (0w ≧ median vs < median ) | 0.628 | 0.289-1.364 | 0.240 |  |  |  |  | |
| erbB-2 (0w ≧ median vs < median ) | 0.885 | 0.404-1.937 | 0.759 |  |  |  |  | |
| HGF (0w ≧ median vs < median ) | 0.727 | 0.340-1.553 | 0.410 |  |  |  |  | |
| sIL-6Rα (0w ≧ median vs < median ) | 0.719 | 0.333-1.553 | 0.401 |  |  |  |  | |
| Leptin (0w ≧ median vs < median ) | 0.447 | 0.202-0.990 | 0.047 |  | 0.311 | 0.111-0.876 | 0.026 | |
| OPN (0w ≧ median vs < median ) | 0.956 | 0.445-2.050 | 0.907 |  |  |  |  | |
| PDGF-AB/BB (0w ≧ median vs < median ) | 0.817 | 0.382-1.748 | 0.603 |  |  |  |  | |
| PECAM-1 (0w ≧ median vs < median ) | 0.804 | 0.376-1.720 | 0.574 |  |  |  |  | |
| PRL (0w ≧ median vs < median ) | 0.907 | 0.424-1.941 | 0.801 |  |  |  |  | |
| SCF (0w ≧ median vs < median ) | 0.840 | 0.394-1.792 | 0.652 |  |  |  |  | |
| sTIE2 (0w ≧ median vs < median ) | 0.932 | 0.432-2.011 | 0.858 |  |  |  |  | |
| sVEGFR-1 (0w ≧ median vs < median ) | 0.985 | 0.454-2.318 | 0.969 |  |  |  |  | |
| sVEGFR-2 (0w ≧ median vs < median ) | 1.193 | 0.559-2.546 | 0.647 |  |  |  |  | |
| Ang2 (0w ≧ median vs < median ) | 1.303 | 0.610-2.785 | 0.495 |  |  |  |  | |
| sCD40L (0w ≧ median vs < median ) | 0.764 | 0.358-1.633 | 0.488 |  |  |  |  | |
| EGF (0w ≧ median vs < median ) | 0.993 | 0.464-2.124 | 0.985 |  |  |  |  | |
| ENG (0w ≧ median vs < median ) | 1.148 | 0.528-2.494 | 0.727 |  |  |  |  | |
| sFASL (0w ≧ median vs < median ) | 1.277 | 0.591-2.758 | 0.534 |  |  |  |  | |
| HB-EGF (0w ≧ median vs < median ) | 1.275 | 0.593-2.741 | 0.534 |  |  |  |  | |
| IGFBP-1 (0w ≧ median vs < median ) | 0.974 | 0.458-2.074 | 0.946 |  |  |  |  | |
| IL-6 (0w ≧ median vs < median ) | 0.782 | 0.361-1.692 | 0.532 |  |  |  |  | |
| IL-8 (0w ≧ median vs < median ) | 1.436 | 0.668-3.086 | 0.354 |  |  |  |  | |
| IL-18 (0w ≧ median vs < median ) | 1.354 | 0.623-2.942 | 0.445 |  |  |  |  | |
| PAI-1 (0w ≧ median vs < median ) | 1.447 | 0.676-3.086 | 0.341 |  |  |  |  | |
| PLGF (0w ≧ median vs < median ) | 0.827 | 0.380-1.801 | 0.632 |  |  |  |  | |
| TGF-α (decreased vs increased) | 1.020 | 0.467-2.225 | 0.961 |  |  |  |  | |
| TNF-α (0w ≧ median vs < median ) | 0.821 | 0.377-1.788 | 0.691 |  |  |  |  | |
| uPA (0w ≧ median vs < median ) | 1.240 | 0.579-2.653 | 0.580 |  |  |  |  | |
| VEGF-A (0w ≧ median vs < median ) | 0.817 | 0.382-1.747 | 0.602 |  |  |  |  | |
| VEGF-C (0w ≧ median vs < median ) | 1.138 | 0.533-2.432 | 0.738 |  |  |  |  | |
| VEGF-D (0w ≧ median vs < median ) | 1.171 | 0.543-2.524 | 0.688 |  |  |  |  | |
|  |  |  |  |  |  |  |  | |
|  |  |  |  |  |  |  |  | |
