# Supplementary material for: Prognostic value of plasminogen activator inhibitor‐1 in biomarker exploration using multiplex immunoassay in patients with metastatic renal cell carcinoma treated with axitinib
Source: Health Sci Rep. 2020 Oct 15;3(4):e197. doi: 10.1002/hsr2.197 (PMC7559632; doi:10.1002/hsr2.197)
Supplement: Supplementary file 6 — Table S4. Cox proportional hazard model to predict the shorter overall survival using baseline clinical parameter and serum biomarker level. [file HSR2-3-e197-s006.docx]

| Supplementary Table 4: Cox proportional hazard model to predict the shorter overall survival using baseline clinical parameter and serum biomarker level. | | | | | | | |
| --- | --- | --- | --- | --- | --- | --- | --- |
|  |  |  |  |  |  |  |  |
| Variable | Univariate analysis | | |  | Multivariate analysis (stepwise) | | |
|  | HR | 95% CI | P value |  | HR | 95%CI | P value |
|  |  |  |  |  |  |  |  |
|  |  |  |  |  |  |  |  |
| Age (< median vs >median) | 0.480 | 0.174-1.324 | 0.156 |  |  |  |  |
| Gender (male vs female) | 0.854 | 0.274-2.658 | 0.785 |  |  |  |  |
| BMI (<25 vs ≧25) | 0.602 | 0.208-1.745 | 0.350 |  |  |  |  |
| Previous treatment (No vs Yes) | 0.534 | 0.182-1.568 | 0.253 |  |  |  |  |
| pT (≧pT2 vs pT1) | 1.233 | 0.386-3.942 | 0.724 |  |  |  |  |
| cN (≧cN1 vs cN0 ) | 4.691 | 1.562-14.089 | 0.006 |  | 2.292 | 0.483-10.883 | 0.297 |
| LVI (Yes vs No) | 1.494 | 0.326-6.853 | 0.606 |  |  |  |  |
| Grade (G2-3 vs G1) | 1.439 | 0.597-3.473 | 0.418 |  |  |  |  |
| Number of metastasis (≧3 vs 0−2) | 4.104 | 1.487-11.321 | 0.006 |  | 2.709 | 0.357-20.533 | 0.335 |
| Lung metastasis (yes vs no) | 0.912 | 0.311-2.674 | 0.867 |  |  |  |  |
| Liver metastasis (yes vs no) | 2.841 | 0.904-8.924 | 0.074 |  |  |  |  |
| Bone metastasis (yes vs no) | 3.255 | 1.198-8.846 | 0.021 |  | 2.472 | 0.370-16.492 | 0.35 |
| CRP (≧ ULN vs < ULN ) | 3.102 | 0.703-13.684 | 0.135 |  |  |  |  |
| Alb (< LLN vs > LLN) | 3.417 | 0.769-15.175 | 0.106 |  |  |  |  |
| Hb (< LLN vs > LLN) | 3.382 | 1.090-10.496 | 0.035 |  | 1.996 | 0.534-7.453 | 0.304 |
| Thrombocyte( <ULN vs ≧ULN) | 3.046 | 0.957-9.699 | 0.059 |  |  |  |  |
| sEGFR (0w ≧ median vs < median ) | 0.557 | 0.206-1.506 | 0.249 |  |  |  |  |
| FGF-basic (0w ≧ median vs < median ) | 1.240 | 0.457-3.364 | 0.673 |  |  |  |  |
| Follistatin (0w ≧ median vs < median ) | 1.174 | 0.423-3.253 | 0.758 |  |  |  |  |
| G-CSF (0w ≧ median vs < median ) | 1.013 | 0.367-2.795 | 0.980 |  |  |  |  |
| erbB-2 (0w ≧ median vs < median ) | 0.973 | 0.352-2.687 | 0.958 |  |  |  |  |
| HGF (0w ≧ median vs < median ) | 0.714 | 0.259-1.971 | 0.516 |  |  |  |  |
| IL-6Rα (0w ≧ median vs < median ) | 0.983 | 0.361-2.673 | 0.973 |  |  |  |  |
| Leptin (0w ≧ median vs < median ) | 0.656 | 0.242-1.780 | 0.408 |  |  |  |  |
| OPN (0w ≧ median vs < median ) | 1.350 | 0.502-3.628 | 0.552 |  |  |  |  |
| PDGF-AB/BB (0w ≧ median vs < median ) | 1.260 | 0.456-3.482 | 0.656 |  |  |  |  |
| PECAM-1 (0w ≧ median vs < median ) | 1.305 | 0.478-3.567 | 0.603 |  |  |  |  |
| PRL (0w ≧ median vs < median ) | 1.095 | 0.410-2.920 | 0.857 |  |  |  |  |
| SCF (0w ≧ median vs < median ) | 0.473 | 0.164-1.364 | 0.166 |  |  |  |  |
| TIE2 (0w ≧ median vs < median ) | 1.010 | 0.365-2.795 | 0.985 |  |  |  |  |
| sVEGFR-1 (0w ≧ median vs < median ) | 1.350 | 0.504-3.621 | 0.550 |  |  |  |  |
| sVEGFR-2 (0w ≧ median vs < median ) | 1.112 | 0.41-2.975 | 0.832 |  |  |  |  |
| Ang2 (0w ≧ median vs < median ) | 2.307 | 0.814-6.540 | 0.116 |  |  |  |  |
| sCD40L (0w ≧ median vs < median ) | 0.763 | 0.284-2.046 | 0.591 |  |  |  |  |
| EGF (0w ≧ median vs < median ) | 1.458 | 0.516-4.125 | 0.477 |  |  |  |  |
| ENG (0w ≧ median vs < median ) | 2.030 | 0.690-5.971 | 0.198 |  |  |  |  |
| sFASL (0w ≧ median vs < median ) | 2.633 | 0.880-7.877 | 0.083 |  |  |  |  |
| HB-EGF (0w ≧ median vs < median ) | 2.161 | 0.753-6.187 | 0.151 |  |  |  |  |
| IGFBP-1 (0w ≧ median vs < median ) | 1.146 | 0.430-3.057 | 0.785 |  |  |  |  |
| IL-6 (0w ≧ median vs < median ) | 1.645 | 0.582-4.645 | 0.348 |  |  |  |  |
| IL-8 (0w ≧ median vs < median ) | 2.480 | 0.835-7.369 | 0.102 |  |  |  |  |
| IL-18 (0w ≧ median vs < median ) | 1.842 | 0.652-5.205 | 0.249 |  |  |  |  |
| PAI-1 (0w ≧ median vs < median ) | 1.515 | 0.580-4.065 | 0.410 |  |  |  |  |
| PLGF (0w ≧ median vs < median ) | 0.983 | 0.356-2.715 | 0.974 |  |  |  |  |
| TGF-α (decreased vs increased) | 2.091 | 0.708-6.173 | 0.182 |  |  |  |  |
| TNF-α (0w ≧ median vs < median ) | 0.966 | 0.350-2.669 | 0.947 |  |  |  |  |
| uPA (0w ≧ median vs < median ) | 2.086 | 0.709-6.137 | 0.182 |  |  |  |  |
| VEGF-A (0w ≧ median vs < median ) | 1.861 | 0.667-5.191 | 0.235 |  |  |  |  |
| VEGF-C (0w ≧ median vs < median ) | 2.306 | 0.824-6.455 | 0.112 |  |  |  |  |
| VEGF-D (0w ≧ median vs < median ) | 1.767 | 0.631-4.945 | 0.279 |  |  |  |  |
|  |  |  |  |  |  |  |  |
|  |  |  |  |  |  |  |  |
